# Salivary inflammatory proteins in patients with oral potentially malignant disorders

**DOI:** 10.4317/jced.55917

**Published:** 2019-07-01

**Authors:** Valentina R. Dikova, Sara Principe, Jose V. Bagan

**Affiliations:** 1 Faculty of Medicine and Dentistry, Valencia University, Fundación Investigación Hospital General Universitari Valencia, Spain; 2 Service of Stomatology and Maxillofacial Surgery, Hospital General Universitari de Valencia, Faculty of Medicine and Dentistry, Valencia University, Spain

## Abstract

Cytokines are a group of small proteins involved in the regulation of infection, immune responses and inflammation. Since altered cytokine responsiveness has been linked to Oral Squamous Cell Carcinoma (OSCC), research to date indicates the possibility of using salivary pro- and anti-inflammatory proteins for screening of oral disorders. OSCC is a multistep neoplasia in which many genetic and epigenetic changes have been correlated to cancerous transformation of oral potentially malignant disorders (OPMD) such as oral leukoplakia, erythroplakia and lichen planus. The goal of the innovative salivary diagnostics is the identification of a single or multiple biomarkers that will serve as a clinical test facilitating the diagnosis of patients predisposed to develop OSCC. Based on scientific literature review, this article summarizes the results from nine articles, all of them being case-control studies where TNF-α, IL-1β, IL-6 and IL-8 were considered possible indicators of malignant transformation.

** Key words:**Interleukins, saliva, oral premalignant lesions.

## Introduction

OSCC represents the most common malignant neoplasm of the oral cavity and it comprises 80-90% of Head and Neck Cancers (HNC). Over the past two decades, the 5-year survival rate remains at about 50% due to its initially asymptomatic nature, leading to advanced stages diagnosis with few therapeutic options. Besides, its tendency to produce regional metastases in the neck, as well as its frequent recurrences after treatments should be taken into account. OSCC tumors appear through a series of molecular mutations leading to uncontrolled cellular growth from hyperplasia areas to dysplastic lesions, to carcinoma in situ and is finally followed by invasive carcinoma. Smoking and drinking alcohol along with environmental and genetic factors are considered major risk factors in the western world, whereas in Asian countries tobacco/areca nut/betel leaf chewing has an important role in the aetiology. Human papillomavirus (HPV) infections have also been reported in those patients but with scarce influence on OSCC located in the anterior part of the mouth (1). Currently, the diagnosis is based on a histopathologic examination of the lesion from an excisional biopsy taken from the suspected area upon direct visual clinical checkup (2). The primary treatment for early stages is a surgical intervention. Radio/chemotherapy is indicated when there are affected margins after the surgical resection or the presence of metastatic lesions observed in more advanced disease cases. Therefore, early detection is crucial for improving the treatment outcomes and the survival rate of the patients. Oral cancer develops through a multistep process whose mechanism of action remains not well understood, but the initial presence of a precursor cell subsequently evolving into cancer has been established (3). A complex of genetic, epigenetic and metabolic changes have been correlated to cancerous transformation of Oral Potentially Malignant Disorders (OPMD) (4). According to the World Health Organization (WHO), lesions and conditions of the oral mucosa, with a predisposition to malignant conversion, are defined as OPMD. Typical representatives are oral leukoplakia, erythroplakia, lichen planus and submucous fibrosis. Determining which OPMD will follow a stable clinical course and which will progress to invasive carcinoma is challenging with the routine histopathological diagnosis and has limited prognostic value. Therefore, the development of alternative methods for predicting malefic potential of suspicious lesions is of high demand. Although several different biological and molecular factors have been proposed as diagnostic tools for oral cancer, there is a long way to go until a real impact on routine clinical care is established. Proteomic and transcriptomic indicators have yielded promising results while information obtained from microorganisms and immunologic factors remain one of the more intriguing aspects in the pursuit of biomarkers, identified from various body fluids (5).

The interest in human saliva has evidently increased due to its advantages, in some cases, over other samples such as blood, urine and exfoliated cells. Saliva comprises a non-invasive, rapid to collect, cost-effective and easy to store biomaterial (6). The array of salivary molecular and microbial analytes (proteins, enzymes, DNA, mRNA, etc.) makes it a significant source of discriminatory, measureable and quantifiable parameters (8-10), such as pro- and anti-inflammatory proteins (cytokines) whose expressions are regulated by the coordinated activation of various signaling pathways as a response to inflammation. An increasing number of investigations report the use of salivary cytokines as diagnostic tool for oral disorders. Recent research has suggested that nuclear factor - κB (NF- κB) dependent cytokine levels are elevated in saliva and tissue specimens of patients with OPMD (9). Thus, cytokines with proinflammatory and proangiogenic activity are thought to be prominent agents in the local and systemic nature of these responses, and a promising tool that could indicate early cancer onset. However, more extensive studies with a larger sample population and sensitivity and specificity of the clinical targets are required to validate prognostic significance.

The main aim of this article is to review the available literature and to summarize the implications of salivary inflammatory proteins for studying OPMD, as well as to comment on their capacity as potential biomarkers for malignant transformation.

## Material and Methods

A bibliographical search was performed on Pubmed, where scientific articles were chosen based on keywords: “salivary interleukins, oral premalignant disorders, oral potentially malignant disorders, oral precancer”.

The inclusion criteria for selecting the articles were:

1. The articles reported research into interleukins in human saliva samples.

2. The methodology of detection and quantification of salivary interleukins was described and validated.

3. The samples were obtained from individuals with OPMD including Oral leukoplakia, Proliferative verrucous leukoplakia and Oral lichen planus.

4. The articles reported detailed results on the interleukins findings, their role in the diagnosis, and their relationship with possible malignant degeneration.

5. All the articles were published in English over the period of the last 15 years.

Finally, nine publications that fulfilled the inclusion criteria were selected and the mean of analyzed variables, such as the investigated inflammatory factors and their importance or role in the diagnosis of malignant transformation, was reported based on descriptive statistical analysis only.

## Results

- Data on the selected articles

All the articles selected for this review were case-control studies, with the oldest dating from 2004 and the most recent from 2016. The publications come from six different countries, namely Croatia, Belgium, Austria, Spain, Japan and India ( Table 1). Saliva samples had been obtained from voluntary study participants − healthy individuals (Controls) and patients, clinically diagnosed with OPMD – who had signed a written consent form approved by the Institutional Ethical Committee of the respective university hospital departments. For comparison and validation of statistical differences between the groups, mainly non-parametric Man-Whitney test for independent samples was performed.

**Table 1:**
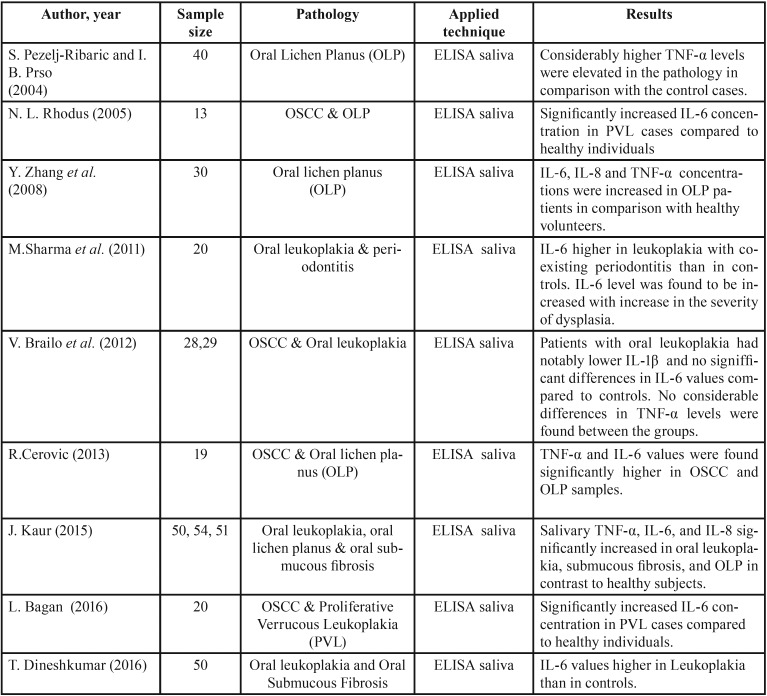
Data on findings of investigated cytokines in OPMDs.

- Data on findings regarding the implication of cytokines for studying OPMD

As inflammation has previously been linked to the pathogenesis of OSCC, multiple reports suggest that Nuclear Factor κB (NF-κB)-dependent overproduction of certain cytokines is similarly observed in patients with OPMD. As the most typical representative, homogeneous leukoplakia has been widely explored and several authors have published case-control studies where Enzyme Linked Immunoadsorbent Assay (ELISA) was used to detect and quantify inflammatory proteins in biofluid samples. Patients with clinically confirmed oral leukoplakia were registered to have significantly higher salivary concentration of Interleukin 6 (IL-6) when compared to healthy individuals (12-14). Besides, Tumor Necrosis Factor alpha (TNF-α) and IL-8 levels were also found elevated in the leukoplakia samples (13,14). In contrast, in 2012, Brailo *et al.* (14) found considerably lower values of salivary IL-1β compared to controls and no differences in IL-6 and TNF-α levels between the same groups. It should be considered that the OPMD group was composed of patients diagnosed with oral leukoplakia without epithelial dysplasia, as well as with mild, moderate and severe epithelial dysplasia, based on histopathological reports. A single study was conducted comparing IL-6 salivary levels in Proliferative verrucous leukoplakia (PVL) and control samples. PVL starts as simple homogenous leukoplakia but progresses to verrucous forms with a high risk of malignant transformation and recurrence after treatment. Notably increased values were registered in the pathology samples in contrast to the control cases (15). Expression patterns of salivary cytokines were investigated in Oral lichen planus (OLP) – a complex local immunologically mediated disease of the oral mucosa. In 2004 a group of authors (16) reported elevated secretion of TNF-α in the reticular and erosive/atrophic forms of OLP. A year later Rhodus (17) published a case-control study with multiple cytokine detection. TNF-α, IL-1α, IL-6 and IL-8 were determined in human spit by ELISA and found significantly increased in patients with clinically confirmed OLP in contrast to healthy subjects. In support of these findings are also other working groups (13,14,19). The potential role of IL-18 as an indicator for OLP diagnosis was shown by Y. Zhang et al (19). Some researchers have reported a correlation between target cytokine abundance and clinico-pathologic features such as size and aggressiveness of the premalignant lesion (14,21); a number of articles also take into account the patients’ smoking habits (12,13,15,21).

## Discussion

Nomenclature, definitions and classification issues of oral precancer were widely discussed in a workshop coordinated by the WHO Collaborating Centre for Oral Cancer and Precancer in the UK in 2005. The consensus views of an expert group were presented in a report reflecting the better understanding of multi-step carcinogenesis in the oral mucosa (21). According to the report, the term ‘potentially malignant disorders’ was recommended to refer to precancer as it conveys that not all disorders described under this term may transform into cancer. However, since then many other publications have analysed the above terms, mainly the definition of oral leukoplakia (22). Based on the epidemiologic research, the epithelial lesion could be the cause of most oral malignancies. Identification of etiological factors and clinical and histopathological methods for diagnosis are in increasing need for prevention and early treatment. Oral epithelial dysplasia (OED) is a histopathological term used to describe tissue changes observed in a chronic, progressive and premalignant disorder of the oral mucosa (23). Moreover, dysplastic changes are consistently seen in the mucosa adjacent to the tumor in patients with invasive OSCC. OED is considered the main histologic marker of possible evolution to malignancy and is thought to be predictive of an increased rate of development of SCC (24). Other techniques for diagnosis of OED and possible malignant potential of oral lesions have been proposed: brush biopsy, liquid-based cytology, toluidine blue, vizilite technique and oral auto fluorescence (25). However, their specificity and sensitivity are not as reliable as the incisional biopsy and histologic method. Oral leukoplakia is noted as the most common premalignant disorder in the oral mucosa with malignant transformation rates varying from 1-17% (26-30). The variety of manifestation forms and potential to convert into SCC has stimulated growing interest in research at cellular and molecular levels. A greater risk are considered to be the non-homogenous forms such as verrucous, nodular (speckled), and erythroplastic types (31,32). Immunological analysis of leukoplakia and OLP specimens reported the presence of different inflammatory cells in connective tissue, which suggests chronic inflammation (25). This ongoing inflammatory response causes progressive damage to the body leading to different diseases. Salivary analytes have already been proved useful for the detection of local, systemic and infectious disorders (33–36). According to the National Institute of Health (NIH), a biomarker is an objectively measured and evaluated indicator of normal biologic and pathogenic processes or pharmacologic responses to therapeutic treatment that exists in a variety of forms including antibodies, microbes, DNA, RNA, lipids, metabolites and proteins (34). In terms of biomarkers for inflammatory response, there is a rise in expression of cytokines. They are secreted from epithelial and immune cells, thereby promoting concentration of macrophages and neutrophils and thus producing inflammation (35). The clinical significance of salivary cytokines has grown since powerful evidence has revealed the critical involvement of NF-κB mechanism of action in carcinogenesis, apoptosis protection and chemoresistance in neoplasia including breast, ovarian, gastric, pancreatic and HNC (39–42). The NF-κB is a key player in inflammatory response and its signaling pathway is largely based on its role in the expression of proinflammatory agents including cytokines such as IL-1, IL-6, TNF-α, chemokines, and adhesion molecules (40). Besides, it has been shown that OSCC cells express increased levels of TNF-α, IL-1α, IL-6 and IL-8 (10,11). The local and systemic nature of these responses suggests the hypothesis that altered proinflammatory cytokine responsiveness is tightly associated with precancer and cancer and could contribute to the pathogenesis of the oral malignancy.

All of the case-control studies described in this review are in support of the hypothesis, demonstrating significant growth of IL-1α, IL-6, IL-8 and TNF-α levels in patients with OPMD when compared to healthy individuals (12-21). The higher rates of NF-κB mediators might be associated with the development of oral precancerous and cancerous lesions. In normal cellular conditions, stimulation with cytokines leads to growth inhibition, whereas in oral cancer cells up regulation of positive cell cycle regulators such as NF-κB and Signal Transducers and Activations of Transcription (STAT) pathway contributes to cell survival and proliferation. Although TNF-α causes necrosis of some types of tumors, it might act as an inducer of tissue remodeling required for tumor growth and spread. IL-6 is a multifunctional cytokine, originally described as a regulator of immune and inflammatory response signaling but also detected in multiple epithelial tumors (43). Hence, elevated secretion of IL-6, IL-8 and TNF-α in OPMD might be indicative for the transition of a lesion from benign into malignant. It is suggested that altered cytokine production and responsiveness in oral cancer takes place primarily in the oral cavity (14). Thus, the search for OSCC biomarkers in saliva becomes an attractive alternative to other biofluids approach due to its direct contact with the oral mucosa and lesions. Furthermore, we should highlight that IL-1β was reported to have the highest salivary values of all studied cytokines in comparison to serum, where the values were below the limit of detection (14). It remains under discussion whether increased salivary cytokines concentration might be a result of a lesion with epithelial discontinuity and surrounding inflammation, not related to cancerogenic conditions. For instance, periodontal diseases can enhance the secretion of several interleukins (44). In addition, correlation studies between IL-6 levels and histopathological grading demonstrated a positive association (11). IL-6 was found to be rising from well differentiating to moderate and poorly differentiating lesions in serum and saliva. These findings suggest this cytokine as a marker that can be associated with the severity and aggressiveness of the disease. Various studies have shown relation of IL-6 with clinical staging in OSCC (42-45). Similarly, histological grading of OPMD has been proposed to influence the levels of salivary cytokines in a growth manner, correlating with the level of epithelial dysplasia (10,14).

## Conclusions

Cytokines are potential key players in salivary diagnostics approaches and currently under investigation as therapeutic targets or agents. Results from diverse studies present evidences that salivary proinflammatory cytokine levels differ significantly between healthy individuals and patients diagnosed with OPMD and OSCC. Thus, they strengthen the potential of these biomolecules to validate their prognostic and/or diagnostic utility. Whether cytokine increase happens before oral cancer becomes clinically evident and whether it could be used for monitoring the malignant transformation of suspicious lesions needs to be further confirmed by larger-scale studies.
